# Epidemiology of acute coronary syndromes in a Mediterranean country; aims, design and baseline characteristics of the Greek study of acute coronary syndromes (GREECS)

**DOI:** 10.1186/1471-2458-5-23

**Published:** 2005-03-16

**Authors:** Christos Pitsavos, Demosthenes B Panagiotakos, Antonis Antonoulas, Spyros Zombolos, Yannis Kogias, Yannis Mantas, Peter Stravopodis, Georgia Kourlaba, Christodoulos Stefanadis, for the GREECS Study Investigators

**Affiliations:** 1 First Cardiology Clinic, School of Medicine, University of Athens, Greece

## Abstract

**Background:**

The present study GREECS was conducted in order to evaluate the annual incidence of acute coronary syndromes (ACS) and to delineate the role of clinical, biochemical, lifestyle and behavioral characteristics on the severity of disease. In this work we present the design, methodology of the study and various baseline characteristics of people with ACS.

**Methods/Design:**

A sample of 6 hospitals located in Greek urban and rural regions was selected. In these hospitals we recorded almost all admissions due to ACS, from October 2003 to September 2004. Socio-demographic, clinical, dietary, psychological and other lifestyle characteristics were recorded. 2172 patients were included in the study (76% were men and 24% women). The crude annual incidence rate was 22.6 per 10,000 people and the highest frequency of events was observed in winter. The in-hospital mortality rate was 4.3%. The most common discharged diagnosis for men was Q-wave MI, while for women it was unstable angina.

**Discussion:**

This study aims to demonstrate current information about the epidemiology of patients who suffer from ACS, in Greece.

## Background

During the past decades epidemiological investigations have provided a portrait of the potential candidate for acute coronary syndromes, in the US, as well as in many other parts of the world, especially in European countries [1-9]. However, many investigators claimed for differences in the profile, i.e. socio-demographic characteristics, prevalence of risk factors, dietary habits, etc. of people who suffer from coronary heart disease, between populations as well as among individuals within populations [10-12]. A potential explanation can be attributed to the gene-environment interactions, as well as various cultural and behavioural particularities.

The profile of cardiovascular disease patients in Greece has been, mainly, investigated from few large scale, population-based studies, like the Seven Countries Study in the early 1960s [13], the Hellenic study of acute myocardial infarction, which recruited about 7500 patients with myocardial infarction from almost all hospitals countrywidethe in early 1990s [14], the CARDIO2000 case-control study of acute coronary syndromes [15], as well as some small case-control or observational studies that included patients from specific areas [16,17]. However, the prevalence, annual incidence and management of patients with coronary heart disease in Greece is unknown, since all these studies recruited patients during certain time periods, and information relating cardiovascular events with treatment, as well as lifestyle habits, like exercise, diet, and psychological stress and depression, are lacking. Additionally, during the past decade, Greece has experienced marked but uneven socio-economic development, with the average income increasing by about 20-fold [18]. Consequently, the lifestyle of people throughout the country has changed dramatically, as well as the incidence of cardiovascular disease.

The aim of the GREEk study of acute Coronary Syndromes (GREECS) is to evaluate the prevalence and annual incidence of acute coronary syndromes (ACS), as well as the characteristics, and management of these patients, in a sample of six Greek urban and rural regions. Secondary goals are to examine the role of adoption to the Mediterranean diet and other lifestyle habits, as well as various clinical, biochemical, psychological and personal characteristics of these patients on the severity and the short (30 days) – long (6, 12 months) – term prognosis. In this work we present the methodology used, and the baseline characteristics of the studied population.

## Methods / Design

### Population of the study

**Figure 1:**
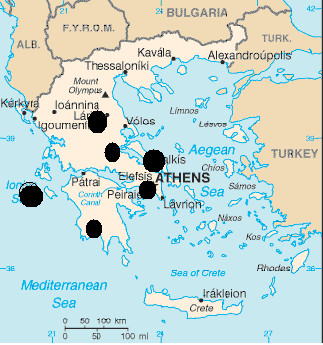
Regions covered by the study.

Between October 1, 2003 and September 30, 2004 (12 months) we enrolled almost all consecutive patients (participation rate = 98%) that entered in the cardiology clinics or the emergency units of six major General Hospitals, in Greece (Hippokration hospital in Athens, and the general hospitals in Lamia, Karditsa, Halkida, Kalamata and Zakynthos island). By the exception of Athens – where there are several other hospitals -, all the other hospitals cover the whole population of the aforementioned regions, including urban and rural areas. The studied regions were randomly selected from all Greek regions in order to cover a wide range of the county (Figure 1).

During the study period 2,172 patients were admitted for ACS in the selected hospitals, 1649 (76%) of them were men and 523 (24%) were women. Power analysis showed that the number of enrolled participants is adequate to evaluate two – sided differences between groups of the study and the investigated parameters greater than 20% (± 5%), achieving statistical power greater than 0.80 at 5% probability level (P-value).

### Diagnosis of ACS

At entry a 12-lead electrocardiogram was performed and clinical symptoms were evaluated in all patients, by a cardiologist of the Study. Based on the electrocardiographic findings patients were classified as having ST-segment elevations, non-ST segment elevations or other electrocardiographic abnormalities. Moreover, blood tests were performed to detect evidence of myocardial cell death. We measured troponin I levels and the MB fraction of total creatinine posphokinase (CPK). According to the Joint European Society of Cardiology and American College of Cardiology Committee, blood samples were obtained on hospital admission, at 6 to 9 h, and again at 12 to 24 h if earlier samples were negative and the clinical index of suspicion was high [19]. We included only cases with discharge diagnoses of ACS (acute myocardial infarction (MI) or unstable angina (UA)). In particular, acute myocardial infarction was defined by at least two of the following features: (a) electrocardiographic changes (patients with or without ST segment elevations), (b) compatible clinical symptoms, and (c) specific diagnostic sensitive biomarkers elevations (troponin I > 0.4 ng/ml and the MB fraction of CPK > 8.8 ng/ml). UA was defined by the occurrence of one or more angina episodes, at rest, within the preceding 48-hours, corresponding to class III of the Braunwald classification [20].

The study was approved by the Medical Research Ethics Committee of our Institution and was carried out in accordance with the Declaration of Helsinki (1989) of the World Medical Association.

### Other clinical and biochemical characteristics

In all patients a detailed medical history was recorded, including previous hospitalization for cardiovascular disease (i.e. coronary heart disease, stroke or other cardiovascular disease), presence and management of hypertension, hypercholesterolemia, renal failure and diabetes mellitus. Moreover, we recorded patients' medical family history. In particular, we asked information concerning first-degree relatives (biological parent, or brother, or sister) about presence of coronary heart disease, hypertension, dyslipidemias and diabetes. Premature history (<55 years old for males and < 65 years old for females) of myocardial infarction, sudden death, coronary arteries bypass grafting procedure or percutaneous coronary angioplasty in first-degree relatives classified the participants in the positive family history group for coronary heart disease [20]. Premature history (<55 years old for males and < 65 years old for females) of hypertension, hypercholesterolemia, hypertriglyceridemia, and diabetes, defined as the use of special medication or known, but untreated, condition classified the participants in the positive family history group for these co-morbidities. In addition to troponin I and the MB fraction of creatinine mentioned above, we also measured white blood cell counts, urea, and uric acid.

### Demographic, anthropometric and lifestyle characteristics

Socio-demographic characteristics included: age, sex, marital status and number of children, years of school, type of occupation and occupational skills, which they were evaluated through a ten-point scale from unskilled – hand workers (lower values) to executive – skilled workers (higher values) that has been developed for the purposes of the Study; and mean annual income of the family (through self reports) during the last three years. Regarding people in the family who were not working, we used the average family income, while for unemployed individuals we used the basic monthly allowance they take from the Social Service Office.

Height and weight was measured, to the nearest 0.5 cm and 100 g respectively. Body mass index (BMI) was then calculated as weight (in kilograms) divided by height (in meters) squared. Based on the World Health Organization [21], overweight was defined as BMI between 25 and 29.9 kg/m^2^, while obesity as BMI greater than 29.9 kg / m^2^.

To evaluate physical activity status of the patients during the past year we used a modified version of a self-reported questionnaire provided by the American College of Sports Medicine [22]. Based on this questionnaire we assessed the frequency (times per week), duration (in minutes per time) and intensity of sports or occupation related physical activity. Participants who did not report any physical activities were defined as sedentary. For the rest of the participants we calculated a combined score by multiplying the weekly frequency, duration and intensity of physical activity.

Current smokers were defined as those who smoked at least one cigarette per day or have stopped cigarette smoking during the past 12 months. Former smokers were defined as those who had stopped smoking more than one year previously. The rest of them were defined as never smokers or rare smokers. Exposure to environmental cigarette smoke for at least 30 minutes per day and three days per week at workplace, home or other public places was recorded in all patients, too.

### Nutritional habits and dietary ascertainment

The evaluation of the nutritional habits was based on a validated semi-quantitative food frequency questionnaire [23]. The consumption of certain food items and the portion size as an average per week, during the past year, was recorded. Then, the frequency of consumption was quantified approximately in terms of the number of times a month the food was consumed. In order to describe total diet composite scores were applied, which are necessary for the evaluation of epidemiological associations. According to a dietary pyramid that has been developed to describe the Mediterranean dietary pattern [24] we calculated a special diet score for each participant that assessed adherence to the Mediterranean diet (range 0 – 55). In particular, for the consumption of 11 items presumed to be close to this pattern (i.e. those suggested on daily basis or more than 4 servings per week) we assigned score 0 when a participant reported no consumption, 1 when reported consumption of 1 to 4 times / month, 2 for 5 to 8 times, 3 for 9 to 12 times / month, 4 for 13 to 18 times / month and 5 for more than 18 times per month. On the other hand, for the consumption of foods presumed to be away from this diet (like meat and meat products) we assigned the opposite scores (i.e. 0 when a participant reported almost daily consumption to 5 for rare or no consumption). Especially for alcohol we assigned score 5 for consumption of less than 3 wineglasses per day, score 0 for consumption of more than 7 wineglasses per day and scores 1 to 4 for consumption of 3, 4 to 5, 6 and 7 wineglasses per day. Higher values of this diet score indicates greater adherence to the Mediterranean diet, while lower values indicate adherence to the "Westernized" diet.

### Psychological evaluation

Depressive symptomatology was assessed using a translated and validated version of the Center of Epidemiological Studies Depression Scale (CES-D) [25]. The CES-D is a well known and world-widely used self-rating scale for the measurement of depression. It is a self-reporting instrument and was originally developed in order to assess depression symptoms without the bias of an administrator affecting the results. Higher scores on this scale are indicative of more severe depression [25]. CES-D consists of 20 items that cover affective, psychological, and somatic symptoms. The patient specifies the frequency with which the symptom is experienced (that is: a little = 1, some = 2, a good part of the time = 3, or most of the time = 4). Previous investigations indicate that the CES-D score is a valid and sensitive measure of clinical severity in depressed patients and support its continued use as a research instrument [26].

Clinical symptomatology of occupational stress was determined by a special self-reported questionnaire based on the survey obtainable by the Job Stress Help Line [27]. By this questionnaire the presence of occupational stress and insecurity was evaluated and summarized for every patient by scoring each item of the questionnaire (1 = yes, 0 = no). Total score ranged from 0 to 10.

### Statistical analysis

In this, brief, baseline report continuous variables are presented as mean values ± standard deviation. The categorical variables are presented as absolute and relative (%) frequencies. Future analyses will follow with appropriate statistical techniques. Associations between continuous variables and group of patients will be evaluated through the analysis of variance, after controlling for equality of variances (homoscedacity) using the F-test. Due to multiple comparisons we will apply the Bonferroni rule to correct for the inflation of type – I error. Associations between categorical variables will be tested by the use of the chi-squared test, without the correction of continuity. Correlations between continuous variables will be tested by the use of Pearson's correlation coefficient for the normally distributed, and by the use of Sperman's rho coefficient for the ordinal or skewed variables. The association between the investigated socio-demographic, clinical and biochemical characteristics on the short term outcome (i.e. 1 month) will be tested by the development of multiple logistic regression models, while the association of the aforementioned characteristics on the long term outcome (6 and 12 months) will be tested by the use of Cox proportional hazards models. Appropriate tests for goodness-of-fit (i.e. deviance and Pearson's residuals) will be applied in all models. All statistical calculations will be performed on the SPSS version 12.0 software (SPSS Inc, Texas, U.S.A.).

### Baseline characteristics

From October 2003 to September 2004, 2172 patients with discharge diagnosis of ACS were enrolled into the study (1649 men, 65 ± 13 years old and 523 women, 62 ± 11 years old, p < 0.001). The men-to-women ratio was 3- to -1. The annual incidence of ACS was 22.6 per 10000 of people (34.0 per 10000 men and 10.9 per 10,000 women). The annual incidence was calculated with the exception of events at Hippokration Hospital in Athens, because it was very difficult to define the referent population since there are many hospitals in the area. The mean number of daily admissions was 6 ± 3 persons per day, averaging 5 ± 4 men and 2 ± 1 women.

**Figure 2:**
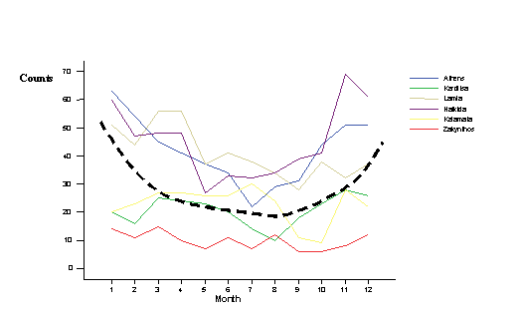
Monthly distribution of hospital admissions for ACS in the centers of the study (dotted line represents the smoothed 12 month average)

Table 1 illustrates the age-sex distribution of the patients. Figure 2 illustrates the series of the monthly number of admissions for ACS in the selected hospitals during the 12 months period. A seasonal variability was observed in the counts of hospital admissions due to ACS (Figure 2).

**Table 1:** Age-sex distribution of the patients

Age (years)	Men (n = 1609)	*Women (508)*
*< 30*	5 (0.3%)	*0 (0.0%)*
*30–39*	28 (1.7%)	*5 (1.0%)*
*40–49*	220 (13.3%)	*31 (5.9%)*
*50–59*	335 (20.3%)	*52 (9.9%)*
*60–69*	388 (23.5%)	*115 (22.0%)*
*>70*	673 (40.8%)	*320 (61.2%)*

Of the 2172 patients enrolled into the study, 38% had ST segment elevations, 27% had non-ST segment elevations and the rest of them, i.e. 35% patients, had other electrocardiographic findings. According to the discharge diagnosis, 764 (35%) patients were diagnosed as having unstable angina, 699 (32%) patients as having non-Q-wave MI and 709 (33%) patients as having Q-wave MI.

Table 2 presents the cross-tabulation of electrocardiographic (ECG) findings and discharge diagnosis. The majority of patients with ST segment elevations had Q-wave MI, while about 61% of patients with an undetermined electrocardiographic pattern had UA. It is of interest that a considerable proportion of patients without ST segment elevations or other ECG findings had Q-wave MI. Moreover, 8% of patients with ST segment elevations were defined as having UA at discharge.

**Table 2:** Electrocardiographic (ECG) findings and discharge diagnosis

	Diagnosis at discharge		
ECG changes	Q-wave MI	Non-Q-wave MI	*Unstable angina*

*ST-segment elevations*	88%	18%	*8%*
*Without ST-segment elevations*	5%	46%	*31%*
*Other ECG findings*	7%	36%	*61%*

% values are by diagnosis group

Table 3 illustrates various baseline clinical characteristics of the patients by discharge diagnosis. We also found that 24% of ACS patients had family history of diabetes, 24% of patients had family history of dyslipidemias and 43% of patients reported family history of hypertension.

**Table 3:** Clinical characteristics by discharge diagnosis

	Unstable angina	Non-Q-wave MI	Q-wave MI
***Men***			
Number	544 (33%)	523 (32%)	582 (35%)
Age (years)	65 ± 12	67 ± 13	63 ± 13
Body mass index (kg/m^2^)	28 ± 4.7	27 ± 3.5	27 ± 37
Obesity (%)	116 (24%)	86 (20%)	113 (22%)
Hypertension (%)	257 (51%)	215 (41%)	215 (41%)
Hypercholesterolemia (%)	245 (49%)	151 (42%)	212 (46%)
Diabetes mellitus (%)	146 (30%)	145 (34%)	134 (27%)
Renal failure (%)	22 (5%)	31 (8%)	23 (6%)
Prior coronary heart disease	299 (60%)	212 (51%)	153 (29%)
***Women***			
Number	220 (42%)	176 (34%)	127 (24%)
Age (years)	70 ± 10	74 ± 11	72 ± 13
Body mass index (kg/m^2^)	28 ± 5.1	28 ± 4.2	28 ± 4.2
Obesity (%)	52 (26%)	27 (20%)	37 (31%)
Hypertension (%)	160 (76%)	106 (71%)	76 (63%)
Hypercholesterolemia (%)	105 (50%)	61 (50%)	52 (46%)
Diabetes mellitus (%)	80 (40%)	63 (45%)	32 (28%)
Renal failure (%)	13 (7%)	17 (13%)	3 (3%)
Prior coronary heart disease	115 (56%)	63 (44%)	29 (25%)

P < 0.05 and **P < 0.01 between diagnosis group after correcting for multiple comparisons through the Bonferroni adjustment

Table 4 illustrates various lifestyle and behavioral characteristics of the patients by discharge diagnosis.

**Table 4:** Lifestyle and behavioral characteristics by discharge diagnosis

	Unstable angina	Non-Q-wave MI	Q-wave MI
***Men***			
Number	544 (33%)	523 (32%)	582 (35%)
Diet score (0–55)	27 ± 2.6	26 ± 2.7	24 ± 2.4
CES-depression score (0–60)	18.5 ± 11	22.5 ± 10	21 ± 10
Physical inactivity (%)	20%	17%	22%
Former smoking (%)	50%	41%	32%
Current smoking (%)	37%	47%	57%
Passive smoking (years)	16 ± 15	17 ± 15	20 ± 16
Financial status			
*Low*	7%	5%	8%
*Medium*	53%	65%	55%
*High*	37%	28%	33%
*Very high*	3%	2%	4%
Years of school	8 ± 4	7.7 ± 4	9.0 ± 4.6
Occupational skills (0–10)	4.2 ± 1.7	4.1 ± 1.4	4.6 ± 1.6
***Women***			
Number	220 (42%)	176 (34%)	127 (24%)
Diet score (0–55)	26 ± 2.6	25 ± 2.4	23 ± 2.3
CES-depression score (0–60)	24 ± 11	23 ± 11	20 ± 9
Physical inactivity (%)	32%	28%	38%
Former smoking (%)	11%	5%	11%
Current smoking (%)	49%	71%	55%
Passive smoking (years)	23 ± 15	22 ± 15	21 ± 16
Financial status			
*Low*	15%	9%	13%
*Medium*	58%	73%	66%
*High*	23%	18%	16%
*Very high*	4%	1%	5%
Years of school	6 ± 4.3	5.5 ± 3.6	6 ± 3.5
Occupational skills (0–10)	4.0 ± 1.3	3.5 ± 1.2	3.8 ± 1.2

* P < 0.05 and **P < 0.01 between diagnosis group after correcting for multiple comparisons through the Bonferroni correction

The median (and 25^th^, 75^th ^percentiles) time between the overt of symptoms and the time medical care was sought, was 4 (2, 10) hours. Based on the discharge diagnosis the duration of hospitalization was 7 (5, 8) days for patients with Q-wave MI, 6 (5, 8) days for non-Q-wave patients and 5 (3, 7) days for UA patients. Furthermore, 60% of patients with ST-elevation received trombolytic therapy.

The in-hospital mortality rate was 36 deaths per 1000 male patients and 63 deaths per 1000 female patients (i.e. overall 82 deaths). The in-hospital mortality rate of patients with ST segment elevation was 74 deaths per 1000 patients, for non-ST segment elevation was 34 deaths per 1000 and for undetermined electrocardiographic findings was 2 deaths per 1000 patients.

## Discussion

In this brief report we presented the design, and the aims of an epidemiological study of ACS, that has been conducted in six major general hospitals, in Greece (i.e. the GREECS). We also presented the baseline characteristics of almost all patients who hospitalized in these hospitals for ACS during the study period (i.e. October 2003 to September 2004).

The overall incidence rate of ACS observed in our survey was 22.6 events per 10000 of population. Based on this figure it could be speculated that the prevalence of ACS in the investigated Greek areas is 2.6%. In a similar recent study located only in northwestern Greece, which also included sudden cardiac death before hospital admission, the incidence rate of ACS was much higher (i.e. 39 events per 10000 people) [17]. Based on a review paper by Chimonas [28] that evaluated the prevalence and incidence of ACS in Greece during 1988, we observe that the current rates are slightly higher in men compare to the annual incidence in late 1980s (i.e. 29.7 events per 10000 of people), but the observed incidence rate in women is almost two-fold as compared to 1988 (i.e. 5.2 events per 10000 people). Future analyses of our study will answer to the questions whether the observed difference in incidence rates could attribute to various lifestyle and behavioral changes, like dietary and smoking habits, or other environmental particularities, occurred in Greece the last years.

We also observed that the annual incidence for men was 34 per 10,000 of people, while the incidence for women was significantly lower, i.e. 11 per 10,000 of people. This finding will be tested whether can be attribute to the increased smoking habits observed in women during the past decades, as well as to various other lifestyle habits and work-related conditions, like physical inactivity, unhealthy diet, social insecurity and job stress, observed in female population from late 1960s to present [18].

Furthermore, we observed that the in-hospital mortality rate in was 4.3%, which was similar to the in-hospital mortality rate that has been calculated based on a sample of 25 European countries, i.e. 4.9% [9].

In this work we have also presented various baseline clinical and lifestyle characteristics of the enrolled patients. Future analyses will evaluate the role of the investigated clinical characteristics as well as family history of the common cardiovascular risk factors on the severity and prognosis of the ACS patients. Regarding dietary and other lifestyle related habits, we aim to evaluate whether the adherence to a Mediterranean dietary pattern, the adoption a physically active lifestyle, the abstinence of smoking and the control of psychological factors is associated with less severe disease and a better short and long term outcome. We anticipate that the completion of the follow up and the analysis of the results will provide current, novel and valuable information about the epidemiology of ACS, in Greece, as well as the role of clinical, psychosocial and lifestyle characteristics on the prognosis of cardiac patients.

## Abbreviations

ACS = acute coronary syndromes

CPK = creatinine posphokinase

MI = acute myocardial infarction

UA = unstable angina

ECG = electrocardiographic

CES-D = Center of Epidemiological Studies depression

BMI = body mass index

## Competing interests

The author(s) declare that they have no competing interests.

## Authors' contributions

CP, DBP = are the principal investigators of the study, had the concept and the design of the study, and wrote the paper, AA, YK, YM, SZ, PS, CS = contributed to the design of the study, GK = contributed to the data management and analysis

## Pre-publication history

The pre-publication history for this paper can be accessed here:


